# Experiences of Female Nurses’ Parental Leave in Taiwan: A Qualitative Study

**DOI:** 10.3390/healthcare11050664

**Published:** 2023-02-24

**Authors:** Ya-Hui Tseng, Kuo-Feng Wu, Hung-Ru Lin

**Affiliations:** 1 School of Nursing, National Taipei University of Nursing and Health Sciences, Taipei City 112303, Taiwan; 2 Department of Business Administration, National Taipei University of Business, Taipei City 100013, Taiwan

**Keywords:** female nurses, parental leave, qualitative study

## Abstract

(1) Background: To counteract the recent severe decline in birthrates in Taiwan, a number of child welfare policies are being promoted. Parental leave is among the most discussed policies in recent years. Nurses are healthcare providers, but their own right to healthcare has not been well investigated and should receive more attention. (2) Aim: This study aimed to understand the experience journey of nurses in Taiwan from considering applying for parental leave to returning to work. (3) Methods: Qualitative design with in-depth interviews was conducted with 13 female nurses from three hospitals in northern Taiwan. (4) Results: Content analysis of the interviews revealed five themes, i.e., considerations for taking parental leave, support received from other parties, life experience during parental leave, concerns regarding the return to the workplace, and preparations for the return to the workplace. Participants were motivated to apply for parental leave due to the lack of help with childcare, the desire to care for their own child, or if their financial situation allowed it. They received support and help during the application process. Participants were happy that they could participate in important developmental stages of their child, but were concerned about disconnect from society. Participants were concerned about not being able to resume work. They successfully returned to the workplace through arranging childcare services, self-adapting and learning. (5) Conclusions: This study can serve as a reference for female nurses considering parental leave and provides insights to management teams for building a friendly nursing workplace and creating mutually beneficial situations.

## 1. Introduction

The parental leave policy in Taiwan states that an employed father or mother who has been covered by labor insurance for more than one year and has a child under the age of three is entitled to parental leave according to the Employment Gender Equality Act, which took effect on 8 March 2002. When applying for parental leave, the parent can apply for a wage subsidy, which is calculated as 60% of the average monthly salary reported on the insurance for the past six months. For each child, the parent may, on an aggregated basis, receive a maximum of six months’ allowance. After July 2021, a new law increased the parental leave subsidy by an additional 20% of the applicant’s average monthly salary. In total, the insured will receive an allowance/subsidy equaling 80% of his/her average monthly salary reported on the insurance [1].

Parental leave is a measure to ensure that new fathers and mothers in the workforce do not lose their jobs when caring for their newborn, and are able to return to their place of work. However, applicants for parental leave may encounter many decision-making situations. Before applying, the couple would have to discuss their family situation. The literature shows the benefits of parental leave, including improved maternal mental health, reduced postpartum maternal depression and intimate partner violence, increased vaccination and breast feeding rates, and reduced infant and child mortality rates. Paid parental leave and the opportunity to be involved in their child’s development are important experiences for a family [2,3,4,5,6].

The availability and generosity of paid parental and home care leave vary considerably across countries. Compared with Taiwan’s 6-month (about 26 weeks) paid parental and home care leave, the Organization for Economic Co-operation and Development (OECD)’s average entitlement available to mothers stands at just above 32 weeks, with most countries that offer at least 1 week providing somewhere between 26 and 52 weeks. However, 11 OECD countries offer no entitlement to paid parental or home care leave at all, while at the other extreme, three OECD countries (Finland, Hungary and the Slovak Republic) provide a statutory entitlement for two-and-a-half-years of paid leave or more [7]. Research suggests that working parents attach great importance to whether the parental leave is paid, and to available childcare arrangements [8,9].

A 2022 Ministry of Labor survey of parents returning to the workplace after taking parental leave showed that only 79% of employees returned to their original workplace in 2019, 73.4% of them had no difficulty in adapting to work after the leave, and 26.6% had difficulty. Women, those working with a large number of employees, and those with a longer average application period were more likely to have difficulties. In that survey, 8.1% reported encountering interference or opposition by their unit or superiors in the process of applying for parental leave; a majority of these (4.8%) reported that they were told that they would be demoted or would be unable to return to their original position [10]. Returning to the workplace after parental leave may also require parents to refresh or update their training. These challenges impose great pressure on employees taking parental leave.

To counteract the recent severe decline in birthrates in Taiwan, a number of child welfare policies are being promoted. Parental leave is among the most discussed policies in recent years. Based on statistical data from the Ministry of Labor in 2022, a total of 626,000 people applied for parental leave up until 2018. Despite equal benefits being available for fathers and mothers, women accounted for the majority of parents taking parental leave (519,000; 82.8%). A total of 88,838 parents took parental leave in 2021, of which 71,335 were women [10]. Furthermore, starting from 1 July 2021, a parental leave wage subsidy has been granted for an additional 20% of the average monthly salary of the insured employee who applies for the parental leave allowance. The Ministry of Labor pointed out that since the implementation of the 20% salary subsidy measure, there are more than 47,000 people who received parental leave allowances from July to December 2021, an increase of more than 11,000 compared with the same period in 2020. About 30% were male applicants, the number of which increased by more than 3700, an increase of 56% [11].

In Taiwan, those who work in a unit with a large number of employees, and who are engaged in medical care, social work, or education are more likely to be obstructed or face difficulties when applying for parental leave [10]. In hospitals, 176,871 (96.2%) registered nurses are female, according to statistics from the Taiwan Union of Nurses Association in 2022 [12]. Female employees are entitled to menstrual leave, job adaptation during pregnancy, adaptive or reduced work time during childcare, and family care leave. However, nurses frequently have a sense of responsibility and are concerned that there will be no one to take over their work in their absence, creating a disincentive to take leave and a further dilemma between work and parenting [13].

Research on parental leave in Taiwan has focused on general employees rather than nurses. Most of the studies focus on the general employees’ willingness to apply for parental leave or the reasons for returning to the workplace. To date, there is no study focused on the experiences from applying for parental leave to deciding to return to the workplace. Not only is the proportion of female nurses much higher than that of male nurses, but women of childbearing age account for 89.3% of total female nurses [14]. Female nurses are caught in a dilemma between not creating extra work for their colleagues and considering their own need to care for a newborn. Nurses are healthcare providers, but their own right to healthcare has not been well investigated and should receive more attention. In this study, we conducted qualitative interviews with female nurses who applied for parental leave to better understand their mental journey throughout the process. The study may serve as a reference for female nurses who are considering taking parental leave and as a workplace guide for nursing management to help create mutually beneficial situations and to promote a friendly and accommodating environment.

## 2. Materials and Methods

### 2.1. Design

A qualitative research design with an in-depth interview method was used to gather information on the subjective aspects of the journey of female nurses in Taiwan from considering applying for parental leave to returning to work. The semi-structured interview guideline (Table 1) was created after discussions with three senior researchers specialized in qualitative study, women’s health, and nursing management.

### 2.2. Pilot Study

Before conducting the data collection, a pilot study was undertaken to confirm the appropriateness of the interview guideline. Two female nurses who have applied for parental leave completed the pilot study. We did not change the interview questions because the participants successfully completed the interview and could fully express their thoughts on parental leave.

### 2.3. Participants and Setting

This study used purposive sampling and recruited female nurses who were willing to share their parental leave experience from two medical facilities and a community hospital in Northern Taiwan. The sampling criteria were as follows: (1) female nurse working at a hospital; (2) have applied for parental leave; (3) able to communicate in Mandarin or Taiwanese; (4) agreed to participate in this study and to be recorded.

### 2.4. Data Collection

The study recruitment period was 1 December 2020 to 30 June 2021. Recruitment notices were posted in the hospital to seek female nurses willing to share their parental leave experience. Researchers informed the participants about the research objectives. Upon filling out the consent form, participants scheduled a one-on-one interview and confirmed an interview location with us. An in-depth interview was carried out based on an interview guide (Table 1). The location for interviews, chosen by the participants, was either in the privacy room at the hospital or at their place of residence, whichever was the most comfortable and relaxing for them. Interviews lasted approximately 45 to 60 min each. During the interview process, the female nurses were encouraged to express their feelings and their experiences. Interviews were audio-taped with the participant’s permission and were transcribed verbatim immediately after the interview. The first author continued to collect data until all the authors were assured that saturation was achieved; that is, data collection was ended when no new themes emerged from the participants’ narratives and the data were redundant [15]. In addition, during each interview, the interviewer recorded the participant’s nonverbal behaviors, any special events that occurred, and thoughts derived from the interview. These observation records were not used in the analysis; however, they helped researchers gain a deeper understanding of the context and meaning of the exchanges in the verbatim text when analyzing the data [16].

### 2.5. Data Analysis

This study used the content analysis method and the Graneheim and Lundman data processing [17]. The analysis steps taken were as follows:

(1) All recorded interview data were transcribed by the first author.
(2) The interview data were read several times to gain a general understanding.
(3) All participants’ perspectives of parental leave were extracted and collated into a single text. The text was sorted into condensed meaning units, abstracted, and labeled with a code.
(4) The various codes were compared based on differences and similarities and sorted into categories and subcategories.
(5) The tentative categories were discussed by all the authors and underwent further revisions.
(6) Finally, the categories’ underlying meaning was formulated into a theme.

### 2.6. Trustworthiness

This study used four criteria proposed by Lincoln and Guba to test the trustworthiness of data collection and analysis [18].

Credibility: Credibility can be increased by choosing an interview location that participants feel comfortable in, ensuring the comprehensiveness and faithfulness of data collection through recording, and interpreting data accurately. Researchers should have undergone training in qualitative research interviews, recording, analyses, and interpretation to enhance their data analysis abilities. Prior to the interview, researchers should introduce themselves via telephone to build a trusting relationship with the participants. This may help participants better express and describe their true feelings. We used the following methods to increase credibility. (a) Peer debriefing: The researcher asked colleagues with similar expertise to analyze two items of information to discover possible alternative interpretations. An experienced researcher was then consulted to guide and answer questions with regards to data analysis. (b) Member checking: The interview was transcribed. The analyzed information was then given to participants, who were asked for their feedback and suggestions.

Transferability: This study used an intentional sampling method to choose participating female nurses from different hospitals and of different educational backgrounds and age to increase our data diversity. The participants’ personal characteristics and subjective feelings were determined distinctly and contemplated repeatedly to examine whether the interview content answers the research questions. These measures contribute to the transferability of the results.

Dependability: The first author conducted all interview to maintain consistency. All recordings, transcriptions, and qualitative coding analyses were retained throughout the process, allowing for an audit trail for purposes of validation of the consistency between the research process and the results.

Confirmability: Researchers remained neutral and objective throughout the study. To prevent loss of information due to blurred memory, audio recordings were transcribed into text within 72 h of the respective interview.

### 2.7. Ethics Considerations

To protect research participants, this study was reviewed and approved by the research ethics committee of a hospital in Northern Taiwan (TCHIRB-10909018-E) before recruitment. All participants entered the study after giving their informed consent and had the right to refuse to participate or withdraw from the study at any time.

## 3. Results

### 3.1. Characteristics of the Participants

We interviewed 13 female nurses aged 28 to 47 (mean age 37.3 years). Educational background ranged from diploma to postgraduate study. The majority had a bachelor’s degree (10; 77%) and worked at a medical center (11; 84.6%). All participants were married (see Table 2).

### 3.2. Themes and Subthemes

We categorized the parental leave experiences of female nurses into five themes: (a) considerations taken when applying for parental leave; (b) support received from other parties; (c) life experience during parental leave; (d) concerns regarding return to workplace; (e) preparations for return to workplace (Table 3).

#### 3.2.1. Considerations Taken When Applying for Parental Leave

Reasons for female nurses to take parental leave included a lack of childcare help from family members, the desire to care for their own child, and a suitable financial situation. These are described in detail below.

##### Lack of Childcare Help from Family Members

Participants applied for parental leave because their family members could not help care for the child. According to participant D, “We have no help and support. We are by ourselves, so it is important to apply for parental leave.” Participant D stated that “We are a small family with just my husband and I. No one can take care of our child, so I thought that I would take a leave to do so.” Participant J said that “My mother-in-law could not take on taking care of my child, so I decided to apply.” Participants proposed to apply for parental leave to care for a newborn due to having only a nuclear family, or because no other family members were able to care for the child.

##### Desire to Raise Their Own Child

Participants wanted to raise their own child and participate in the child’s developmental journey and thus decided to apply for parental leave. Participant B said that “I want to take care of my child and watch my child grow! I just thought that this was an amazing experience!” A participant pointed out that having a child is not just about giving birth but about accompanying the child though childhood. This is an important process of parenting. For example:

My husband and I both think that if we have a child, we need to care for her. We want to raise her ourselves. We try raising her ourselves. I think it’s best if we take care of her ourselves! We can also share this memory together.

(G)

##### Suitable Financial Situation

Participants discussed their financial abilities to support the decision to take parental leave with their husbands prior to applying. For example:

When I was applying for parental leave, income was a consideration I took into account. I calculated whether our money would be sufficient to last until I went back to work. My husband said that if we don’t experience financial pressure, I should take a longer leave until our child is 3 years old.

(J)

#### 3.2.2. Support from Other Parties

Support from other parties when applying for parental leave included support received from family and from superiors at work, and tangible assistance in the form of wage subsidies, as described below.

##### Support from Family

Support from the family was present if the participant discussed the matter with her family before applying for parental leave and received support from family members. For example:

I discussed it with my husband! Even my husband wanted to take a leave and care for our child! My parents supported my decision very much. They think that I should have the experience raising my child and have a shared memory with my kid!

(G)

When my older sister gave birth, the policy did not exist. When I was applying, my mom thought that it would be nice! She is very supportive. She thinks that it’s good!

(K)

##### Support and Assistance from Superiors

This was present if participants brought up their intention of applying for parental leave and received support and assistance from their superiors at work. Some participants also stated that their superior showed support and understanding by empathizing from a mother’s perspective.

Our unit has always been short of staff. I’m also worried that shift arrangements cannot be made if I take a leave. But my superior supported me. She didn’t stop me because of work! She knows that we love children and understands that raising our own children is better. Her kids were raised by her mother-in-law. If she could do it again, she would want to do it herself. She would raise her children because that way her children may be closer to her. She said that now that her two kids are older, they are slightly closer to her mother-in-law. She just thinks that the kids are not as close to their mother.

(E)

I work in the gynecology and pediatrics unit, so my superior actually supported my decision. She is obviously aware of the shortage of staff. I’m part of this team and I am taking a parental leave when there is a staff shortage, I feel bad for my colleagues! But my superior thinks that I should not give up family for work. She’s very supportive and proactively in helping me complete the application.

(F)

##### Tangible Assistance in the Form of Wage Subsidies

Parental leave policy supports participants in the application process. The policy mandates payment of 60% of the average insurance-reported salary for the past six months, starting from the month of the application. Wage subsidies were then given monthly for six months. Participant C said that “This is the government’s policy for us to focus on caring for our child”. Participant K stated that “The parental leave policy is a government policy. It helped us. At least life won’t be too hard with 60% of our salary”. According to participant L, “We get 60% of our salary for the first six months! This is a good intention from the government as the birth rate has been so low recently! They need to figure out a way!” The policy provides women with this paid parental leave in the hope that this may increase birth rates.

#### 3.2.3. Life Experience during Parental Leave

After applying for parental leave, participants had mixed feelings about family life. They were happy that they could participate in every important developmental stage of their children, but also worried that they would be disconnected from society and might feel trapped by having to care for their children all day.

##### Happy to Participate in Every Important Developmental Stage of Their Children

Participants stated that having a chance to be with their children and participate in the different early childhood stages meant a lot to them.

Children only grow up once and every child’s experience is different! They go from first looking at you, to slowly making noises, then to staring at you and even smiling, to the moment they go from crawling to standing up and walking. These are things I want to experience!”

(A)

I recorded when she had her first tooth. I marked it down in my computer when she said something special. I wrote down anything special she said when she started babbling. You don’t just give birth to her and feed her. You need to interact with her, be there with her every step along the way to make her feel secure. You can really understand her behaviors and where she is in the developmental stage.

(I)

##### Trapped in a Situation of Always Caring for a Child

During parental leave, some participants’ daily life shifted to only caring for children, and they felt trapped at home.

During parental leave, I’m always taking care of my children at home. Your life bubble becomes very small. You can’t wander off somewhere like you could at work or after work. Sometimes I want to go back to work to do something different. After half a year, I don’t get 60% of my salary. I realized that I am just spending money and I should go back to work. I need to face the reality! Plus, working is actually easier than taking care of children. You no longer have to care for them on your own.

(K)

I think working is a different kind of relaxing because taking care of children is so tiring! And working is a way to relieve some of the pressure! At work you can take a pause in your role as a mother. You are still you. You are working and you are the previous you at work. Only…only that people know that you have children. You are blablabla’s mother! Yes! You don’t have a name now! I get more satisfaction through working. When raising a child, it is hard to have that satisfaction! You can control many things at work! But you can’t with children! Working diverts your attention from your role as a mother.

(M)

##### Concerns about Being Disconnected from Society

Participants took care of their children at home and rarely interacted with others. They were worried that their personal growth was stagnating. For example:

For me personally, your personal growth stops. You are stuck for months or even a year. You always feel like you are only there to take care of the children. You are always with your children. Unlike working, there you are always doing something; but taking care of children and being with them when they grow, for you, you are not taking a step forward. Your children grow, but you don’t grow. You are on your own and you feel like you can’t take a step in other areas too. You’re stuck with taking care of children!

(A)

I feel ok going back to work! It feels like you take a step forward from the previous feeling of being stuck! You feel less disconnected from society. It’s new and exciting to be with your colleagues. There’s some change and you don’t stagnate. And you don’t feel like you are not contributing to society and the country.

(B)

#### 3.2.4. Concerns about Returning to the Workplace

Nurses returning to the workplace after parental leave experienced uneasiness, including worries that they could not resume previous work or that their children would not be able to adapt to a new care situation.

##### Worry about Not Being Able to Resume Previous Work

Participants pointed out that during their parental leave, new work routines, instruments, or staff might have been introduced at work, requiring them to readjust to a new environment. They were thus concerned that they might have become “rusty” and less familiar with work and that this would affect their work performance. For example:

Leaving work for some time, I am scared that I might be rusty! Many work routines may change. I am worried that I would have to adapt to them.

(A)

I was worried coming back. Work content may be different. Many procedures or details at work might have changed. I have been disconnected with work for some time, I have doubts that I may not be able to resume my previous work and become a problem for others!

(J)

##### Worry That Children Cannot Adjust to a New Care Situation

A return to work requires a change in initial childcare conditions. Children would thus have to face new living situations, caregivers, and environments. Some participants felt guilty and worried that their children might not be able to adjust. For example, participant G said that “We have been together for some time. The sudden separation and not having her around, I am actually really reluctant. I questioned myself whether I am doing the right thing.”

It is a struggle, being separated from your child every day. You see how young she is. And she was crying because you were going to work. You feel torn and question yourself if this is the right thing to do. But you cannot not go to work. It’s a dilemma! Children also need to adapt. You have to consider not only yourself but them too. You will have to consider whether you will have to suddenly switch to a different caregiver for your child again.

(J)

#### 3.2.5. Preparation for Returning to the Workplace

Participants made preparations to resume work, including arrangements for childcare services and for their own adaption and further education.

##### Arranging Childcare Service

A return to the workplace required arranging childcare services. For example, this might consist of having a family member help take care of their children, employing a nanny, daycare, kindergarten, etc.

Before taking the parental leave, I had an idea about when I would return, so I visited some daycare centers at the time. I sent him to daycare a week before I went back to let him adapt to the new environment.

(E)

Before returning to work, my kid will have to…. We have to let her attend a kindergarten. She will have to adapt and get used to the new lifestyle.

(F)

##### Adaptation and Education to Return to Work

Based on the work situation and professional needs at the hospital unit, participants had to adapt and learn in order to return to work.

I spend some time at first adjusting to the changes in some work routines. Then I learned new things. During this time, there may be some new instruments, work routines… you have to learn and adapt to the change.

(J)

I tried not to sleep late. The kid’s daily routine is ok. We, adults, need to adjust. I need to stay awake and maintain a good mental state for work.

(K)

## 4. Discussion

Our investigation of female nurses’ experience in applying for parental leave illuminated a number of considerations that participants had to take into account, and that they received support from multiple parties. These factors affected their parental leave experiences. Considerations regarding the lack of childcare assistance from family members and financial situation are consistent with previous findings [19]. These authors reported that the motivations behind female healthcare workers applying for parental leave were “lack of people to take care of child”, “60% wage subsidy during the leave”, and “wanting a short vacation”. Participants in our study affirmed the importance of the wage subsidy measure during parental leave, suggesting its importance as a factor for two-income families. However, they valued accompanying their child during development more than the subsidy. We found that one of the reasons for taking parental leave was that participants wanted to be with their children during every step of their early life. The children with parents who took parental leave showed better development. Parents with a higher level of education apply for parental leave at a higher rate; these parents are more likely to use multiple resources to stimulate child development [20].

Moreover, parental leave promotes the health of both the mother and the newborn, reducing infant mortality and increasing vaccination and breastfeeding rates [21,22]. In a study assessing childcare responsibility in female healthcare workers, for over 50% of participants, the child was taken care of by family members, and 15% of participants arranged childcare services [23]. The choice of childcare method is made based on the financial situation of the family. It has also been reported that there exists a conflict between childcare and work, and that employment policies such as flexible hours or comprehensive childcare at work are the best methods to facilitate employees’ return to the workforce after parental leave [24].

A study investigating the leadership behaviors of participants’ work superiors showed that if nurses feel that they receive respect, care, and empathy from their superiors, they rated their leadership highly, which in turn has positive effects on the operation of the unit [25,26,27]. Superiors of nursing units have a responsibility to help their colleagues understand paternal leave policies and to educate and support them, in the interest of maintaining a positive and friendly working culture.

We found that female nurses had mixed feelings about their lives during parental leave. They were happy that they could participate in every step of their child’s early development, but worried about becoming disconnected from society due to being with their children all day. Parental leave reduces work hours of parents, increases time with their newborn, and enhances parenting [20]. Although being a parent adds meaning to life, it also introduces burdens and responsibility, reducing free time and in turn affecting quality of life [28]. Fatigue and sleep deprivation, social isolation, and loneliness hinder parents’ adjustment to their new roles. Emotional discord can arise if a mother experiences contentment and affection when caring for the child but the child’s need and her own life come into conflict [29]. Mothers carry out a significantly larger amount of parenting than fathers, and mothers who took parental leave participated even more in parenting [30]. This suggests that women tend to be the long-term care provider for the family regardless of whether they have taken parental leave. Every woman makes different choices between their role as a mother and as an employee. People with high professional expectations may choose to return to the workplace if they see the return as a relief from parenting or if a family member can assist with childcare. On the other hand, some may choose to be full-time mothers if their financial situation, employment conditions, or childcare arrangements allow this [31]. Balancing work and family, sharing responsibilities, and planning parental leave are changes encountered when becoming a parent.

About 68% of parents who had taken parental leave returned to the workplace. Factors enabling their decision to return included “finance”, “adequate care for child”, “concerns about disconnecting from society”, and “collegial relationships”; negative factors included “unable to accommodate work hours (shift work)”, “wanting to be with children”, “no one to care for children”, and “unable to return to previous work unit” [14].

We found that female nurses’ decision to return to the workplace was negatively correlated with childcare pressure and family conflict but positively correlated with work flexibility. Flexibility in shifting work hours and balancing family and work were important for female nurses. Nurses often needed to re-familiarize themselves with work routines after returning. If the workplace could formulate a plan to assist parental leave returnees in adapting to their new work, this may be of substantial benefit [32].

Work–life balance for nurses after returning to the workplace may be negatively affected by fatigue and parenting pressures, and positively affected by the degree of support from colleagues. Health conditions also influence this balance. Thus, collegial relationships and support from superiors are key for a successful return to work. These factors can reduce work–life conflict, enable job satisfaction, and lower emotional pressure [33,34,35].

### Limitations

This study focused on the experiences of female nurses; however, an increasing number of male nurses are joining the workplace, and their experiences may be different. Future studies are required to understand male nurses’ paternal leave experience, which would allow the conclusions to be extended to all nurses and provide a basis for more generally applicable recommendations. In addition, in this study, we only interviewed female nurses who had taken parental leave, but nurses who had children who did not apply for leave or nurses who chose not to have children may have different experiences. Future studies are recommended to understand their experiences or perspectives regarding taking parental leave.

In this study, we strove to consider the age and seniority distribution. Although the results of the study seemed saturated, they were confined to the participants in three hospitals in northern Taiwan. We recommend that researchers conduct interviews in other locations, such as southern or eastern Taiwan, to ascertain whether geographical factors influence the experiences with taking parental leave.

## 5. Conclusions

We found that the main factors affecting female nurses’ decision to take parental leave consisted of the suitable financial situation of the family, the availability of family support, employment conditions, childcare resources, and relevant welfare policies. The main areas of concern voiced in interviews consisted of deliberate considerations prior to taking parental leave, availability of support from multiple parties, conflicting life experiences during parental leave, worries about returning to the workplace, and preparations for the return. Participants experienced many changes and challenges throughout the process. The availability of six months of a 60% salary subsidy provided by the governmental childcare and parental leave policy helps to balance childcare and work and ameliorates financial issues. If superiors in the hospital work unit provide support and assistance from the employee’s perspective, a supportive workplace environment can be created that can help balance employees’ work and family responsibilities and to retain talent. Formulating measures to help female nurses adapt to new work responsibilities upon return also reduces connected anxieties. A comprehensive childcare policy may support female nurses in returning to the workplace without concerns. The findings can serve as a reference for female nurses who are considering taking parental leave and as a workplace guide for nursing management to help create mutually beneficial situations and to promote a friendly and accommodating environment for new and future mothers.

## Figures and Tables

**Table 1:** Interview guide.

1.	Can you talk about the circumstances under which you decided to apply for parental leave?
2.	Who did you talk to before making this decision?
3.	Can you talk about the process of applying for parental leave with the hospital?
4.	What was your living situation after applying for parental leave?
5.	How did you feel about life during parental leave?
6.	Before the end of parental leave, what did you do to prepare for returning to work?
7.	What was your emotional response to returning to the workplace?
8.	How long has it been since you returned to work? What is your living situation now?

**Table 2:** Characteristics of participants.

Participants	Age	Birth Parity	Years Licensed as an RN	Education Level
A	47	2	23	Master
B	31	2	8	Bachelor
C	39	1	16	Bachelor
D	41	1	19	Bachelor
E	28	2	5	Bachelor
F	40	2	16	Associate
G	33	1	11	Bachelor
H	32	2	10	Bachelor
I	41	2	17	Bachelor
J	39	2	11	Bachelor
K	43	2	21	Bachelor
L	36	2	14	Bachelor
M	35	1	12	Associate

Note: Birth parity is defined as the number of times a woman has given birth, both live births and stillbirths at viable gestational age, and “the order refers to the numerical order of the live birth or fetal death”.

**Table 3:** Themes and subthemes.

Themes		Subthemes
1. Considerations taken when applying for parental leave	1-1	Lack of childcare help from family members
	1-2	Desire to raise their own child
	1-3	Suitable financial situation
2. Support received from other parties	2-1	Support from family
	2-2	Support and assistance from superiors
	2-3	Tangible assistance in the form of wage subsidies
3. Life experience during parental leave	3-1	Happy to participate in every important developmental stage of their children
	3-2	Trapped in a situation where of always caring for a child
	3-3	Concerns about being disconnected from society
4. Concerns regarding return to workplace	4-1	Worry about not being able to resume previous work
	4-2	Worry that children cannot adjust to a new care situation
5. Preparations for return to workplace	5-1	Arranging childcare service
	5-2	Adaptation and education to return to work

## Data Availability

The datasets used and/or analyzed during the current study are available from the corresponding author (H.L.) on reasonable request.
